# Early Life Intervention in Paediatrics Supported by E-Health (ELIPSE)—a coaching app for parents to reduce obesity and second-hand smoke exposure in children: study protocols for two parallel-group randomised controlled trials

**DOI:** 10.1186/s13063-025-09251-5

**Published:** 2025-11-18

**Authors:** Alexander Koch, Andrea Wyssen, Clemens Haupt, Johanne Hammelbeck, Hendrika Anette van Dorland, Christa E. Flück, Philipp Latzin, Christoph Saner, Julian Jakob, Michael Kaess, Matthias Volkmar Kopp

**Affiliations:** 1 Department of Paediatrics, Inselspital, Bern University Hospital, University of Bern, Bern, Switzerland; 2 Graduate School for Health Sciences, University of Bern, Bern, Switzerland; 3 University Hospital of Child and Adolescent Psychiatry and Psychotherapy, University of Bern, Bern, Switzerland; 4 Division of Paediatric Endocrinology, Diabetology and Metabolism, Department of Paediatrics, Inselspital, Bern University Hospital, University of Bern, Bern, Switzerland; 5 Department of Biomedical Research, University of Bern, Bern, Switzerland; 6 Division of Paediatric Respiratory Medicine and Allergology, Department of Paediatrics, Inselspital, Bern University Hospital, University of Bern, Bern, Switzerland; 7 Institute of Primary Health Care (BIHAM), University of Bern, Bern, Switzerland

**Keywords:** Childhood, Non-communicable diseases, Obesity, Overweight, Second-hand smoke exposure, Asthma, App, E-Health, Cognitive behavioural therapy

## Abstract

**Background:**

Family lifestyle factors—such as physical activity, diet and parental smoking—are key modifiable determinants for child health. Physical inactivity and calorie-dense diets contribute to obesity, which is linked to 75% of all non-communicable diseases (NCDs). Second-hand smoke (SHS) exposure significantly increases the risk of respiratory NCDs in children, including asthma. Cognitive behavioural therapy (CBT) has proven effective in modifying harmful parental behaviours, but it remains unclear whether app-based CBT for parents can improve family behaviours and child health outcomes. The Early Life Intervention in Paediatrics Supported by E-Health (ELIPSE) project aims to implement and evaluate personalised, family-centred, guided e-health interventions delivered via the ELIPSE mobile app.

**Methods:**

In two randomized controlled trials (ELIPSE I and II), the ELIPSE app will deliver CBT elements, tailored thematic content, and personalised feedback, all remotely guided by a coach. In ELIPSE I, 148 parent–child dyads (child age 6–12 years, BMI > 97th percentile) are randomly assigned 1:1 to treatment as usual (TAU; control group) versus TAU plus full access to the activated ELIPSE app (intervention group). The primary endpoint is the change in children’s ratio of total energy intake to total energy expenditure (TEI/TEE). Secondary endpoints include obesity severity and cardiometabolic risk factors. In ELIPSE II, 160 dyads of a smoking parent and child (< 6 years) are randomly assigned 1:1 to receive either a leaflet on protecting children from SHS exposure (control group) or access to the activated ELIPSE app (intervention group). The primary endpoint is the change in children’s urinary cotinine levels. Secondary endpoints include parental measures to reduce SHS exposure. The intervention period for both trials is 20 weeks, with outcomes assessed at baseline, post-intervention, and 48-week follow-up.

**Discussion:**

The ELIPSE project offers a novel approach to addressing key modifiable NCD risk factors within the family environment, with the goal to improve long-term child health outcomes and reducing morbidity and healthcare costs. Unlike traditional care, the ELIPSE app overcomes time and space constraints, allowing continuous support and personalised guidance.

**Trial registration:**

Both trials prospectively registered at ClinicalTrials.gov: ELIPSE I as NCT06208345 (15 January 2024); ELIPSE II as NCT06311162 (7 March 2024).

**Supplementary Information:**

The online version contains supplementary material available at 10.1186/s13063-025-09251-5.

## Background

Non-communicable diseases (NCDs), such as cardiovascular diseases, type 2 diabetes or chronic obstructive pulmonary diseases, are the leading cause of death worldwide [1]. NCDs often take decades to develop and are strongly driven by modifiable behavioural factors found in early childhood [2, 3]. Major modifiable risk factors for NCDs include obesity, which is associated with 75% of NCDs [4], and second-hand smoke exposure (SHS) [5].

Obesity in children and adolescents is associated with cardiometabolic risk factors including dyslipidaemia, hypertension and impaired glucose metabolism, all of which increase the risk of early morbidity and mortality [6, 7]. Obesity, according to the energy imbalance hypothesis [8], is caused by excessive total energy intake (TEI) at a given total energy expenditure (TEE) [9]. An average reduction in TEI of 110–165 kcal/day may be sufficient to prevent excessive weight gain over time [10]. However, Western diets, with readily available, low-cost, calorie-dense ultra-processed foods specifically marketed at children, make it difficult for families to maintain a balanced diet. Additionally, 80% of school-aged adolescents do not meet the World Health Organization (WHO) guidelines for physical activity [11]. Consequently, one in three European children is overweight or obese [12], and approximately 80% of children with obesity will remain obese until mid-adulthood [13].

SHS exposure increases the risk of respiratory diseases in children. In Europe, about 2–13% of four-year-olds are estimated to have asthma [14]. SHS exposure is associated with an increased risk for wheezing, potentially preceding the manifestation of asthma [15]. It increases the risk for hospitalisation due to asthma exacerbation by twofold [16]. Childhood-onset asthma may persist into adulthood in approximately 40% of cases [17]. Additionally, SHS exposure in children is associated with adverse health effects such as sudden infant death syndrome, impaired lung growth and function, recurrent respiratory infections, obesity and hypertension [18–20]. Given the significant public health and economic burden of childhood obesity and SHS exposure, innovative interventions in early life targeting these modifiable risk factors are essential.

Mobile app-based, family-centred e-health interventions may offer an opportunity to reduce risk exposures for both parents and children while promoting healthier behaviours [21, 22]. A systematic review has shown that particularly game-based approaches promote healthy eating habits and improve nutrition knowledge in children and adolescents. However, only two of the 34 studies included in the review explicitly focused on parent-targeted interventions to improve their children’s health. Evidence for long-term effects of e-health interventions on physical activity and BMI remains limited [23].

Cognitive behavioural therapy (CBT)-based approaches have proven effective in addressing harmful behavioural patterns within families [24]. CBT includes core elements such as psychoeducation, self-monitoring, behavioural activation and problem solving [25]. Studies applying conventional CBT in adults have demonstrated positive effects on weight [26], not only for participants but also for their family members (i.e. for their spouse and children) [27]. Internet- and app-based programs that include guided CBT have shown promise in treating mental disorders and promoting healthy lifestyles [21, 22, 28–30], with outcomes comparable to conventional (i.e. face-to-face) therapies [31–33]. In Sweden, the MINISTOP trials applied app-based CBT to promote physical activity and healthy eating. While these trials showed good behavioural adoption, they did not show significant changes in fat mass or BMI [34, 35]. Notably, children participating in the MINISTOP trials were healthy overall, and less than 5% of them had obesity. In terms of SHS exposure, there are promising long-term results from the FRESH trial, in which CBT elements were delivered through home visits and telephone sessions over 16 weeks, leading to sustained reductions in children’s urinary cotinine levels at 12-month follow-up [36]. Although a small number of apps implementing CBT approaches for smoking cessation are available, none have been tested in a randomised controlled trial or have focused on children’s SHS exposure [37].

Despite some evidence supporting the effectiveness of both CBT and app-based interventions for improving children’s health, there is a notable lack of randomised controlled trials evaluating app-based CBT, particularly for addressing childhood obesity and SHS exposure—two major public health challenges. To address this gap, the ELIPSE project aims to develop, implement and evaluate a personalised, family-centred, guided app-based e-health intervention tailored to reduce childhood obesity (ELIPSE I) and SHS exposure (ELIPISE II).

## Methods/design

The reporting of the ELIPSE study protocols complies with the “Standard Protocol Items: Recommendations for Interventional Trials (SPIRIT)” 2025 guideline (see Additional file 1) [38].

### Study setting and design

**Fig. 1:**
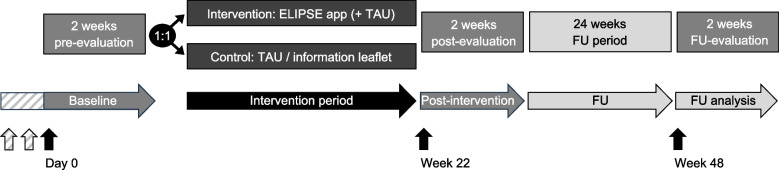
Study design of the ELIPSE I (obesity) and ELIPSE II (smoke) trials. Vertical arrows indicate study visits at day 0 (baseline), week 22 (post-intervention) and week 48 (follow-up). There are two additional baseline visits in ELIPSE I (striped arrows). The ELIPSE app is installed at day 0 and participants of both groups are instructed to document their child’s physical activity and dietary habits (ELIPSE I) or their own smoking behaviour (ELIPSE II) using a basic version of the ELIPSE app during the initial 2-week pre-evaluation period (same in the post- and follow-up evaluation periods). Participating parent–child dyads are randomized 1:1 to either the control or the intervention group. In ELIPSE I, children receive treatment as usual (TAU, control group) or TAU plus access to the full version of the ELIPSE app with additional modules and coaching features (intervention group). In ELIPSE II, participants receive a leaflet on protecting children from SHS exposure (control group) or the full version of the ELIPSE app (intervention group). TAU, treatment as usual; FU, follow-up

The ELIPSE project comprises two single-blind randomised controlled parallel-group trials conducted at the University Children’s Hospital Bern, Inselspital Bern. Psychologists (referred to as coaches) from the University Hospital of Child and Adolescent Psychiatry and Psychotherapy, University of Bern, will provide remote coaching of participants via the ELIPSE app. Participants are randomised in a 1:1 ratio to either the control group or the intervention group (see the ‘Randomisation, blinding procedure and study discontinuation’ section). Data collection time points are outlined in Fig. 1. In brief, baseline (day 0), post-intervention (week 22) and follow-up (week 48) visits are each followed by a 2-week period during which participant-reported outcome measures (PROMs) are collected from both parents and children. All participants are provided with a basic version of the ELIPSE app, used solely for documentation of PROMs during evaluation periods. During the intervention period, participants in the control group of ELIPSE I receive treatment as usual (TAU), which includes lifestyle and dietary counselling at the outpatient weight management service. The control group in ELIPSE II receives a general information leaflet on protecting children from SHS exposure—chosen as a minimal yet ethically appropriate comparator to ensure that all families receive basic health advice. Only participants allocated to the intervention groups in both trials receive access to the full version of the ELIPSE app, either in addition to TAU (ELIPSE I) or in place of the information leaflet (ELIPSE II) (see the ‘Description of the intervention’ section).

### Aims

The aims of ELIPSE I are to:

1. Assess the efficacy of an e-health intervention for parents in reducing their child’s TEI/TEE ratio.
2. Assess the efficacy of an e-health intervention in:
  Reducing the severity of obesity;
  Improving cardiometabolic health (e.g. blood pressure, arterial stiffness, lipid measures);
  Reducing obesity-related chronic low-level inflammation in children;
  Changing parental behaviour in a manner that positively influences their child’s physical activity, diet, and quality of life.
3. Assess the acceptance of the e-health intervention by participants.
4. Assess the sustainability of the intervention on TEI/TEE and severity of obesity at follow-up.

The aims of ELIPSE II are to:

1. Assess the efficacy of an e-health intervention for parents in reducing children’s urinary cotinine levels.
2. Assess the efficacy of an e-health intervention in changing parental smoking-related habits, including adoption of protective measures.
3. Assess feasibility and acceptance of the e-health intervention.
4. Assess the efficacy of an e-health intervention in reducing the frequency and severity of respiratory disease episodes and parental smoking intensity.

In addition, both ELIPSE trials include selected psychological and behavioural screening instruments assessing parental impulsivity, stress, depression, anxiety, emotion regulation, and parenting behaviour, alongside additional measures of smartphone use and working alliance during the intervention (see Table 2). These instruments were chosen to explore potential mediators and moderators of intervention effects and to inform intervention tailoring.

### Participants and recruitment

For ELIPSE I we plan to include 148 dyads of a parent/legal guardian and a child with obesity, and for ELIPSE II we plan to include 160 dyads of a child with a smoking parent/legal guardian (see the ‘Sample size and statistical methods’ section for more details). The inclusion and exclusion criteria are listed in Table 1.

**Table 1:** Inclusion and exclusion criteria of ELIPSE I and ELIPSE II trials

	ELIPSE I (obesity)	ELIPSE II (smoke)
Inclusion criteria	▪ Children aged 6 to ≤ 12 years	▪ Children younger than 6 years
	▪ Children with an age- and sex-matched BMI > 97th percentile according to Swiss national growth charts [39]	▪ Children exposed to second-hand smoke at home (≥ 1 parent/legal guardian smoking tobacco cigarettes)
	▪ Children attending the outpatient weight management service at the University Children’s Hospital Bern	
	▪ Children should live/grow-up in the same household as the parental participant	
	▪ German speaking parent	
	▪ All sex and ethnic backgrounds	
Exclusion criteria	▪ Syndromic obesity^a^	▪ Participation in an active smoking cessation programme
	▪ Known congenital disease affecting ▪ musculoskeletal, cardiac or pulmonary function	
	▪ Participation in another study/trial targeting similar objectives	

^a^ Syndromic obesity encompasses conditions such as Prader-Willi syndrome, Bardet-Biedl syndrome or Alström syndrome[40]

For ELIPSE I, recruitment is conducted through the outpatient weight management service at the University Children’s Hospital Bern. For ELIPSE II, participants are recruited from inpatient and outpatient clinics at the University Children’s Hospital Bern, referrals from other paediatric hospitals, primary care paediatricians, and health care providers in the German-speaking parts of Switzerland, as well as through public outreach via flyers and advertisements on social media platforms and public transport. These materials were either approved by the ethics committee or strictly based on approved templates as specified in the study protocol. To encourage participation and reduce attrition, study visits in ELIPSE II are also offered remotely by phone.

### Randomisation, blinding procedure and study discontinuation

Study participants are randomly assigned in a 1:1 allocation ratio. In ELIPSE I, stratified block randomisation is applied according to the severity of obesity, whereas in ELIPSE II, simple block randomisation is applied to ensure balanced sample sizes throughout the trial. The random allocation sequence is generated and stored within REDCap (Research Electronic Data Capture, version 15.0.11) [41]. Randomisation is single-blinded in both trials, with study investigators blinded to the allocation sequence. Participants cannot be blinded as it is obvious if one has an activated ELIPSE app or not. Unblinding may be required in emergency situations or in case of adverse events and will be reported to the sponsor-investigator.

Withdrawal from the study by either the parent/legal guardian, the child or both may happen at any time without providing a reason. However, an interview to assess reasons for discontinuation will be offered. In case of adverse events, which may interfere with the study outcomes, the sponsor-investigator may decide to exclude the affected participants.

Minimum app engagement criteria are set for participants in the intervention group to define adequate exposure to the intervention. These include at least one diary entry, one message to the coach, watching one educational video and interaction with the app at least once every two weeks. Participants who do not meet the minimal engagement requirements will not be excluded but will be contacted by the coach to ensure that they understand the tasks, assist them in navigating the app, and motivate active participation. The sponsor-investigator may terminate the study prematurely due to insufficient recruitment, ethical or safety concerns, or changes in accepted clinical practice.

### Description of the ELIPSE app

The ELIPSE app, developed at the University Hospital of Child and Adolescent Psychiatry and Psychotherapy Bern, is a German-language application implemented on the ReconWell platform (ReconWell GmbH, Munich, Germany), which is also responsible for its technical maintenance and support. The ELIPSE app is designed according to evidence-based recommendations for mental health smartphone applications [42]. To ensure relevance and usability, healthcare professionals and a focus group comprising parents were consulted during app development, representing active patient and public involvement. Elements of ‘Design Thinking’—a user-centred methodology increasingly recognised as best practice for e-health development—were applied. This methodology involves understanding user needs (‘empathise’), defining challenges and functions (‘define’), generating and prototyping engaging features (‘ideate’ and ‘prototype’), and testing in real-world settings (‘test’) [43].

While the overall structure of the ELIPSE app is consistent across both trials, the content and features are tailored to trial-specific objectives. Both apps are native (i.e. not web-based) and compatible with Android and iOS. All data is anonymised during processing, and communication with servers is encrypted for data protection (see Additional file 3 for technical details).

### Description of the intervention

**Fig. 2:**
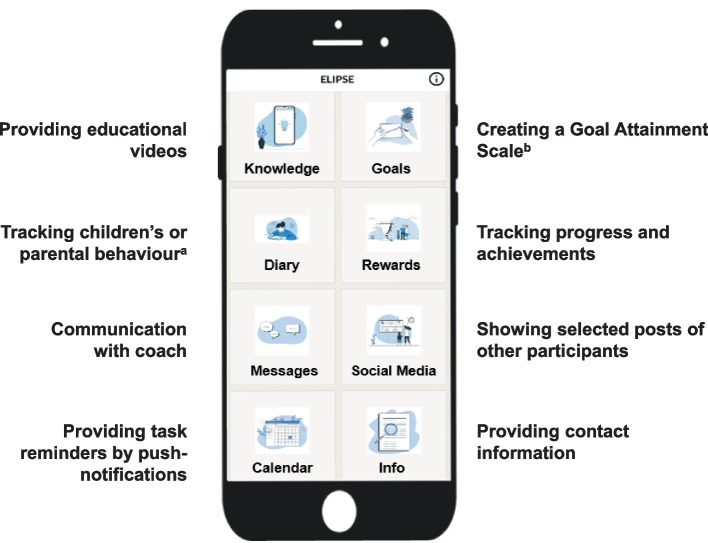
Home screen view with modules of the ELIPSE app (translated from German). ^a^ Tracking children’s physical activity and diet (ELIPSE I) or parental smoking behaviour (ELIPSE II). ^b^ A Goal Attainment Scale measures progress toward personalised goals using a predefined scale of expected outcomes

The ELIPSE app consists of several modules and features (see Fig. 2). The basic version, provided to all participants at baseline, includes access only to the calendar, the info module with contact details, and the diary module for documentation of children’s physical activity and dietary habits (ELIPSE I) or parental smoking behaviour (ELIPSE II) during evaluation periods (see Additional file 3 for an overview of PROMs). Participants in both intervention groups are subsequently granted access to the additional modules and coaching features for the 20-week intervention period. A coach ‘behind the app’ (i.e. a trained psychologist) provides structured personalised feedback, instructions and support up to three times per week via chat function (‘Messages’), which can also be used by participants to communicate with the coach.

The main CBT elements implemented in the app include psychoeducation (via educational videos), behavioural activation and problem-solving (via coaching and goal setting) as well as self-monitoring (via diary entries). The app uses automated (e.g. push notifications and reminders), semi-automated (e.g. graphical feedback and instructions depending on monitored data) and tailored interventions (e.g. individual instructions for behavioural change). None of the educational or therapeutic modules will be activated for controls during the intervention period.

### Study endpoints and assessments

An overview of endpoints and assessments for ELIPSE I and II is shown in Table 2 (see also Additional file 2 for SPIRIT figures). In ELIPSE I, the primary endpoint is the change in TEI/TEE ratio in children between baseline and post-intervention. TEI will be assessed by collecting 24-h dietary recalls, which have shown good accuracy for assessing nutritional intake in children and adolescents [44]. For each evaluation period (baseline, post-intervention, and follow-up), three 24-h recalls are conducted: two on weekdays and one on a weekend day. Recalls will be collected using interviews conducted by a study nurse trained by a paediatric dietitian. One parent and the child together will be asked to provide the recall, to minimise the rate of under-reporting or misreporting [45]. A multiple-pass 24-h recall method will be used, which includes three distinct passes to collect information about a child's food intake during the preceding 24 h: (i) the parent is openly asked to recall everything the child ate during the previous day using any recall strategy; (ii) the parent is asked to clarify any foods mentioned in the first pass (e.g. if breakfast cereals were mentioned in the first pass, the study nurse would then ask whether milk was added, and if so, the type and amount thereof); (iii) the study nurse reviews the list of foods mentioned and probes for additional eating occasions and clarifies food portion sizes.

**Table 2:** Description of study endpoints and assessments for both ELIPSE trials

Endpoint	Assessments [time points]^a^
*ELIPSE I (obesity)*	
* Primary endpoint*	
Change in TEI/TEE ratio	24-h dietary recalls [44, 45] and bioimpedance analysis [49] [baseline, post-intervention, follow-up]
* Secondary endpoints*	
Change in severity of obesity	BMI z-score, %BMIp95, percentage body fat and waist circumference [baseline, post-intervention, follow-up]
Change in cardiometabolic risk factors	Ambulatory blood pressure monitoring [50], carotid-femoral pulse wave velocity [51], fasting (8-h fast) venous blood sample (i.e., lipid profile, glucose tolerance) [baseline, post-intervention, follow-up]
Change in chronic low-level inflammation	Inflammatory biomarkers (i.e., hs-CRP, WBC and biomarkers included in targeted metabolomic and proteomic profiles) [52] [baseline, post-intervention, follow-up]
Change in children’s dietary habits	CEBQ [53] and DEBQ^b^ [54] questionnaires, app-based diary^c^ [baseline, during intervention^d^, post-intervention, follow-up]
Change in children’s physical activity	App-based diary^c^, triaxial accelerometery [55] and hand grip strength [56] [baseline, during intervention^d^, post-intervention, follow-up]
Change in parent-reported quality of life in children	KidScreen-27 questionnaire [57] [baseline, post-intervention, follow-up]
* Tertiary endpoint*	
Acceptance and usability of the app	MAUQ [58] and app follow-up (satisfaction with app modules) questionnaires [post-intervention]; Frequency and duration of app-use, dropout rate [baseline, during intervention^d^, post-intervention, follow-up]
ELIPSE II (smoke)	
* Primary endpoint*	
Change in children’s SHS exposure	Urinary cotinine levels^e^ [baseline, post-intervention, follow-up]
* Secondary endpoints (child)*	
Change in protective measures taken by parents to protect children from SHS exposure	Questionnaire^f^ and app-based diary^c^ [baseline, during intervention^d^, post-intervention, follow-up]
* Tertiary endpoint*	
Acceptance and usability of the app	MAUQ [58] and app follow-up (satisfaction with app modules) questionnaires [post-intervention]; Frequency and duration of app-use, dropout rate [baseline, during intervention^d^, post-intervention, follow-up]
* Quaternary endpoint*	
Frequency of airway disease episodes in children	Questionnaire^f^ [baseline, post-intervention, follow-up]
Use of inhalant medications or antibiotics for airway disease in children	Questionnaire^f^ [baseline, post-intervention, follow-up]
Frequency of severe airway disease episodes in children requiring hospitalisation	Questionnaire^f^ [baseline, post-intervention, follow-up]
Change in parental smoking intensity (number of cigarettes smoked per day)	Questionnaire^f^ and app-based diary^c^ [baseline, during intervention^d^, post-intervention, follow-up]
Additional psychological/behavioural screening tools (ELIPSE I and ELIPSE II)	
Parental impulsivity	BIS-15 questionnaire [59] [baseline]
Parental stress level	PSS-10 questionnaire [60] [baseline]
Parental symptoms of depression	PHQ-9 questionnaire [61] [baseline]
Parental anxiety symptoms	GAD-7 questionnaire [62] [baseline]
Parental emotion regulation	DERS-16 questionnaire [63] [baseline]
Parenting behaviour	DEAPQ-EL-GS questionnaire [64] [baseline, post-intervention, follow-up]
Smartphone usage	MTUAS questionnaire [baseline]
Working alliance	WAI-SR [65] [during intervention]

*TEI* total energy intake, *TEE* total energy expenditure, *BMI* body mass index, *%BMIp95* percentage of the 95th BMI percentile for age and sex, *hs-CRP* high-sensitivity C-reactive protein, *WBC* white blood count, *CEBQ* Children’s Eating Behaviour Questionnaire, *DEBQ* Dutch Eating Behaviour Questionnaire, *MAUQ* mHealth App Usability Questionnaire, *BIS-15* Barratt Impulsiveness Scale, *PSS-10* Perceived Stress Scale, *PHQ-9* Patient Health Questionnaire, *GAD-7* Generalized Anxiety Disorder Assessment, *DERS-16* Difficulties in Emotion Regulation Scale, *DEAPQ-EL-GS* German extended version of the Alabama Parenting Questionnaire for primary school children, *MTUAS* Media and Technology Usage and Attitudes Scale, *WAI-SR* Working Alliance Inventory short revised

^a^ Baseline and post-intervention assessments are the primary focus for efficacy evaluation, while follow-up assessments examine the sustainability of observed effects

^b^ The Dutch Eating Behaviour Questionnaire (DEBQ) assesses parental eating behaviour and was included as a potential mediator of change in children’s eating behaviour

^c^ Items and prompts included in the diary modules of ELIPSE I and ELIPSE II can be found in Additional file 3

^d^ ‘During intervention’ applies only to app-based assessments in the intervention group

^e^ Urine samples from inpatient children will only be considered if admission and collection was on the same day. Otherwise, urine will be collected by parents after children have returned to home environment for at least one week and sent to study site

^f^ Questionnaires related to parental smoking behaviour and child respiratory health can be found in Additional file 3

Food portion sizes include common household measures and dishes such as grams (for weighed ingredients), tablespoons, or cups. Nutritional data will be analysed and micro- and macronutrients calculated using the PRODI software (version 7.0.0.1; Nutri-Science GmbH, Freiburg, Germany). TEI (in calories), weight (in grams) and proportional energy intake from fats, carbohydrates, and proteins will be calculated for each day.

TEE will be calculated using bioimpedance-related body composition measures (e.g. lean and fat mass in kilograms) according to formulas by Pontzer et al. [46]. Bioimpedance will be measured using a multi-frequency bioelectrical impedance analyser (InBody770, InBody Co., Ltd., Seoul, Republic of Korea). It has been validated in paediatric cohorts and provides accurate estimates of body composition, similar to those by dual x-ray absorptiometry (DXA) [47].

For ELIPSE II, the primary endpoint is the change in children’s urinary cotinine levels between baseline and post-intervention. Urinary cotinine reflects SHS exposure in previous days with a median half-life of approximately 28 h [48]. Urine samples will be collected at baseline, post-intervention and follow-up and stored at − 20 °C until further processing. Urine sampling is done using one of the following methods: paediatric urine bag, cotton wools placed in infants’ diapers, clean catch, or collection from a clean cup for toddlers. Urinary cotinine (normalised to creatinine) will be analysed using liquid chromatography coupled to tandem mass spectrometry [51].

### Adverse events

We do not expect any serious adverse effects (i.e. life-threatening or requiring hospitalisation) from participation in either of the trials. Adverse events of interest to be recorded include headache from using the app, emerging parent–child conflicts, negative emotional reactions to messages, psychological distress from self-observation or general study requirements, unexpected physical or emotional reactions to self-defined goals, cognitive fixation on health, negative emotional reactions after group allocation, and stress from the withdrawal of support by the coach after completing the intervention or privacy breaches. All adverse events are collected, fully investigated, followed up until resolution, and documented in REDCap.

Participants are further instructed at baseline to report any app malfunctions directly to the coaching team via the app to enable timely troubleshooting and continuous system improvements. Such technical issues are managed and documented separately by the app development team.

### Sample size and statistical methods

For ELIPSE I, the calculated sample size to detect a difference of 150 kcal/day in energy intake or energy expenditure, at type I error rate of α = 0.05 and power = 0.8 (β = 0.2), implying a moderate effect size of Cohen’s d = 0.45, is *n* = 128 (at a mean TEI of ~ 1700 kcal/day). Considering a dropout rate of 15% [66], we plan to include 148 participants (74 per group).

For ELIPSE II, the minimum total sample size required to detect a 50% reduction in cotinine levels, with a type I error rate of α = 0.05, power = 0.8 (β = 0.2) and an effect size of Cohen’s d = 0.34, is *n* = 114 [67]. The minimum total sample size to detect difference in hospitalisation for asthma in children exposed to SHS exposure is *n* = 136, based on published odds ratio of 1.85 (95% CI 1.20–2.86) [16]. To account for the latter and assuming a dropout rate of 15% [66], we plan to include 160 participants. Participants who dropout before 10 weeks of observation will be replaced by recruiting new participants in both trails.

Descriptive statistics will be used to summarise baseline characteristics for demographic variables and all primary and secondary endpoints. Results will be provided as mean and standard deviation for continuous variables and in number and percentage for categorical variables. Independent sample t-tests will be used to compare mean values for endpoints between groups. Multiple linear and logistic regression models will be fitted for outcomes as dependent variables and group allocation as an independent variable. Variables will be adjusted for potential confounders. Sensitivity analyses will be performed to investigate the robustness of the results to different assumptions and analyses. Missing outcome measurements will be imputed using the Multiple Imputation by Chained Equations (MICE) method [68]. We will use Inverse Probability of Censoring Weights (IPCW) to account for participants lost to follow-up. All statistical analyses will be performed using R or STATA by qualified study team members and verified by a designated biostatistician. A *p*-value of 0.05 will be considered statistically significant. No interim analyses are planned for either trial.

### Dissemination plans

The results of the ELIPSE studies will be published in peer-reviewed scientific journals and presented at scientific conferences [69]. A lay summary of the trial results will be published on the Swiss Clinical Trials Portal Human Research Switzerland (HumRes) to inform the public and study participants.

## Discussion

By tackling key modifiable risk factors in childhood—obesity and SHS exposure, the ELIPSE project aims to prevent NCDs at their origin. Within two randomised controlled trials, we assess the efficacy of a novel app-based e-health intervention. The ELIPSE app integrates expertise from healthcare professionals in medicine and psychology and addresses harmful family behaviours using a CBT approach tailored for parents. It incorporates key CBT elements (i.e. psychoeducation, problem-solving and self-monitoring). Our vision is to offer an accessible e-health intervention that empowers parents to adopt healthier behaviours, ultimately improving child health outcomes.

Recruiting parents for e-health interventions involving their children is a challenge. However, for ELIPSE I, early experiences of recruitment at the weight management service reveal an overall high level of motivation to participate. This may be because most patients attending the weight management service have a long-standing history of obesity or overweight and are actively seeking medical help after recognising the need for intervention. Also, many children and families already suffer from the detrimental physical and social effects accompanying the condition. Although general readiness to make changes among parents of children with excess weight is reported to be high, readiness varies significantly when it comes to implementing specific lifestyle changes related to physical activity and diet [70]. In another study, parents reported that the lack of external support, lack of concrete recommendations, and missing contact persons to discuss practical problems were barriers to making lifestyle changes [71]. With ELIPSE I, we may close this gap and provide much-needed guidance and support. However, in the event of delayed recruitment progress, increasing clinic capacities to accommodate referred patients from waiting lists more promptly and implementing flexible scheduling may support participation rates and sustain enrolment.

For ELIPSE II, recruitment may pose a bigger challenge. When it comes to SHS exposure, parents may not perceive the issue as immediately pressing or may not consider smoking cessation. Studies have found that smoking parents are less aware of the risks that SHS exposure poses for children and are less likely to adopt avoidance behaviours [72, 73]. In a small study in Brazil, 52% of smokers did not consider their children to be second-hand smokers and did not believe that exposure could have adverse health effects on them [74]. A general disinterest in participating in a research study and concerns regarding time requirements and commitment could be additional challenges, as highlighted by a study that explored reasons that parents gave for not participating in a smoking cessation trial [75]. Moreover, traditional approaches usually focus on smoking cessation, which may overwhelm parents and appear unachievable. A study among smoking fathers of newborns showed that both the attempt and success rates were substantially higher for adopting no-smoking-at-home rules (78% attempted, 60% achieved) than for quitting smoking altogether (20% attempted, 4% achieved) [76]. Despite high proportions of parents expressing willingness to quit smoking (57% of parents with hospitalised children and 23% of parents with children in outpatient clinics [77]), targeting smoking cessation alone may not be the most effective strategy. We propose that the focus on lowering SHS exposure and the personalised approach of ELIPSE II increases parents’ readiness to engage in behaviours that prioritise their children's health. In case of substantial recruitment barriers, strengthening collaborations with other paediatric hospitals and increasing in-person recruitment by staff directly engaging with potential participants may help parents modify their beliefs and enhance participant referrals and enrolment.

A challenge for both trials might be study attrition related to app usage and problems with adhering to app content and recommendations of the coach during the 20-week intervention period [78]. Our monitoring of the participants’ frequency and duration of app usage and the guidance by the coach will allow us to provide motivational input to mitigate attrition and promote long-term engagement.

The duration of the 20‑week intervention in the ELIPSE trials warrants consideration. For ELIPSE I, the intervention length is close to the median duration of 20.7 weeks calculated from a meta‑analysis of behavioural and psychological interventions targeting childhood and adolescent obesity [79]. Interventions lasting 6–12 months showed slightly more favourable effects on anthropometric measures. However, the continuous availability of the ELIPSE app and its integration within the outpatient weight management service may help offset this slightly shorter intervention duration. A limitation remains that individual variation in response and the persistence of behavioural changes could mean that post‑intervention and follow‑up assessments do not necessarily capture the maximum possible effect for each participant. For ELIPSE II, a meta‑analysis of smoking‑cessation interventions for parents found that most programmes consisted of brief interventions with only a few individual sessions (e.g. via telephone counselling, face‑to‑face meetings or home visits) [80]. In contrast, ELIPSE II offers sustained and easily accessible support for smoking parents over a 20‑week period.

The current ELIPSE app requires trained psychologists to provide coaching to parents. Our goal is to standardise coaching interventions to support wider dissemination and streamline future implementations. Building on the experiences from ELIPSE I and II, we plan to evaluate further options including live coaching (just-in-time interventions), full automation or an Artificial Intelligence (AI)-guided app. The potential of AI-guided apps may be substantial, offering scalable and tailored support. A digital AI-driven e-health intervention effectively helped participants lose an average of 14% of their body weight over 24 weeks [81]. However, AI-guided e-health interventions in their current form are primarily based on computing probabilities, but essential coaching skills—such as genuine empathy, moral reasoning and creativity—remain uniquely human.

Following the ELIPSE I and II trials, we plan to extend our e-health intervention to address other target populations or modifiable behavioural risk factors, i.e. women who smoke during pregnancy, smoking adolescents, adolescents and young adults with obesity and children with type 1 diabetes or other chronic disorders. Based on our results, the e-health intervention will be evaluated for its adoption and generalisability to other medical settings and regions in Switzerland and internationally.

In summary, the ELIPSE project aims to develop, implement and evaluate a personalised, family-centred, guided app-based e-health intervention to increase physical activity and healthy eating in families with children with obesity and reduce SHS exposure of children with smoking parents. The ELIPSE project provides significant potential for implementation and adaptability to different populations at risk, with the goal of reducing the negative impacts of NCDs on public health and healthcare spending.

### Trial status and duration

Recruitment for ELIPSE I and ELIPSE II commenced in February and May 2024, respectively. Recruitment for ELIPSE I is expected to last until February 2027, with a corresponding study end date in January 2028. The last patient for ELIPSE II is expected to be recruited in January 2027, with a study end date in January 2028.

## Supplementary Information

Additional file 1. SPIRIT checklist. Completed SPIRIT checklist for ELIPSE I and II

Additional file 2. SPIRIT figures. Schedule of assessments and procedures for ELIPSE I and II

Additional file 3. Extended methods. 3a: Technical details of app development and platform. 3b: ELIPSE I (obesity) diary content. 3c: ELIPSE II (smoke) diary content. 3d: ELIPSE II (smoke) questionnaires on parental smoking behaviour and child respiratory health.
Additional file 4. Ethical approval document. Confirmation by the Cantonal Ethics Committee of Berne, Switzerland.
Additional file 5. Funding documentation. Confirmation of peer-review and funding information

## Data Availability

Original protocols for both trials are available upon request from the corresponding author. Following study completion, the data will be made available upon reasonable request, at the discretion of the sponsor-investigator, and subject to ethical approval and data-sharing agreements.

## Abbreviations

AI: Artificial intelligence
BMI: Body mass index
CBT: Cognitive behavioural therapy
NCD: Non-communicable disease
PROM: Participant reported outcome measure
SHS: Second-hand smoke
TAU: Treatment as usual
TEI: Total energy intake
TEE: Total energy expenditure
