# Supplementary material for: Early Life Intervention in Paediatrics Supported by E-Health (ELIPSE)—a coaching app for parents to reduce obesity and second-hand smoke exposure in children: study protocols for two parallel-group randomised controlled trials
Source: Trials. 2025 Nov 18;26:520. doi: 10.1186/s13063-025-09251-5 (PMC12625043; doi:10.1186/s13063-025-09251-5)
Supplement: Supplementary file 1 — Additional file 1. SPIRIT checklist. Completed SPIRIT checklist for ELIPSE I and II [file 13063_2025_9251_MOESM1_ESM.docx]

**Additional file 1** Completed SPIRIT checklist for ELIPSE I & II.

| **Section / Topic** | **No** | **SPIRIT 2025 checklist item description** | **Reported on page no.** | |
| --- | --- | --- | --- | --- |
|  |  |  | **ELIPSE I** | **ELIPSE II** |
| Title and structured summary | 1a | Title stating the trial design, population, and interventions, with identification as a protocol | Page 1, lines 1–3 | |
|  | 1b | Structured summary of trial design and methods, including items from the World Health Organization Trial Registration Data Set | Page 2, lines 43–70; and on ClinicalTrials.gov | |
| Protocol version | 2 | Version date and identifier | Page 2, lines 69–70; page 17, lines 468–471 | |
| Roles and responsibilities | 3a | Names, affiliations, and roles of protocol contributors | Page 1, lines 5–21; page 18, lines 508–513 | |
|  | 3b | Name and contact information for the trial sponsor | Page 1, lines 7, 9, and 16–17; page 18, line 510 | |
|  | 3c | Role of trial sponsor and funders in design, conduct, analysis, and reporting of trial; including any authority over these activities | Page 18, lines 503–506 | |
|  | 3d | Composition, roles, and responsibilities of the coordinating site, steering committee, endpoint adjudication committee, data management team, and other individuals or groups overseeing the trial, if applicable | Page 17, lines 482–491 | |
| **Open science** | | | | |
| Trial registration | 4 | Name of trial registry, identifying number (with URL), and date of registration. If not yet registered, name of intended registry | Page 2, lines 69–70 | |
| Protocol and statistical analysis plan | 5 | Where the trial protocol and statistical analysis plan can be accessed | Full protocol versions (including statistical analysis plan) are not publicly available but can be accessed upon request from the study investigators | |
| Data sharing | 6 | Where and how the individual de-identified participant data (including data dictionary), statistical code, and any other materials will be accessible | Page 16, lines 449–452 | |
| Funding and conflicts of interest | 7a | Sources of funding and other support (e.g., supply of drugs) | Page 17, lines 456–459; and Additional file 5 | |
|  | 7b | Financial and other conflicts of interest for principal investigators and steering committee members | Page 17, lines 453–454 | |
| Dissemination policy | 8 | Plans to communicate trial results to participants, healthcare professionals, the public, and other relevant groups (e.g., reporting in trial registry, plain language summary, publication) | Page 13, lines 341–344 | |
| **Introduction** | | | | |
| Background and rationale | 9a | Scientific background and rationale, including summary of relevant studies (published and unpublished) examining benefits and harms for each intervention | Pages 3–4, lines 74–125 | |
|  | 9b | Explanation for choice of comparator | Page 5, lines 141–143 (treatment as usual was chosen as comparator; no further explanation provided since considered common standard) | Page 5, lines 143–145 |
| Objectives | 10 | Specific objectives related to benefits and harms | Pages 5–6, lines 161–170 | Page 6, lines 172–178 |
| **Methods: Patient and public involvement, trial design** | | | | |
| Patient and public involvement | 11 | Details of, or plans for, patient or public involvement in the design, conduct, and reporting of the trial | Page 8, lines 228–229; page 13, lines 357–358 | |
| Trial design | 12 | Description of trial design including type of trial (e.g., parallel group, crossover), allocation ratio, and framework (e.g., superiority, equivalence, non-inferiority, exploratory) | Pages 4–5, lines 131–146; and Fig. 1 | |
| **Methods: Participants, interventions, and outcomes** | | | | |
| Trial setting | 13 | Settings (e.g., community, hospital) and locations (e.g., countries, sites) where the trial will be conducted | Pages 4–5, lines 131–147 | |
| Eligibility criteria | 14a | Eligibility criteria for participants | Page 7, Table 1 | |
|  | 14b | If applicable, eligibility criteria for sites and for individuals who will deliver the interventions (e.g., surgeons, physiotherapists) | Pages 4–5, lines 133–135 | |
| Intervention and comparator | 15a | Intervention and comparator with sufficient details to allow replication including how, when, and by whom they will be administered. If relevant, where additional materials describing the intervention and comparator (e.g., intervention manual) can be accessed | Pages 8–9, lines 239–261 | |
|  | 15b | Criteria for discontinuing or modifying allocated intervention/comparator for a trial participant (e.g., drug dose change in response to harms, participant request, or improving/worsening disease) | Pages 7–8, lines 215–217 | |
|  | 15c | Strategies to improve adherence to intervention/comparator protocols, if applicable, and any procedures for monitoring adherence (e.g., drug tablet return, sessions attended) | Page 8, lines 217–219 | |
|  | 15d | Concomitant care that is permitted or prohibited during the trial | Page 7, Table 1 | |
| Outcomes | 16 | Primary and secondary outcomes, including the specific measurement variable (e.g., systolic blood pressure), analysis metric (e.g., change from baseline, final value, time to event), method of aggregation (e.g., median, proportion), and time point for each outcome | Pages 9–10, lines 263–294; and Table 3 | |
| Harms | 17 | How harms are defined and will be assessed (e.g., systematically, non-systematically) | Page 12, lines 320–330 | |
| Participant timeline | 18 | Time schedule of enrollment, interventions (including any run-ins and washouts), assessments, and visits for participants. A schematic diagram is highly recommended (see Figure) | Additional file 2 | |
| Sample size | 19 | How sample size was determined, including all assumptions supporting the sample size calculation | Page 12, lines 333–336 | Pages 12–13, lines 337–341 |
| Recruitment | 20 | Strategies for achieving adequate participant enrollment to reach target sample size | Page 6, lines 190–191; page 14, lines 378–380 | Pages 6–7, lines 191–197; page 14, lines 396–399 |
| **Methods: Assignment of interventions** | | | | |
| Randomization: | | | | |
| Sequence generation | 21a | Who will generate the random allocation sequence and the method used | Page 7, lines 205–206 (clarification: the random allocation sequence is generated automatically by REDCap; in practice, allocation within REDCap is visibly only to a designated study team member who is not otherwise involved in recruitment, interventions, or data analyses; this team member then retrieves the corresponding app activation code (for the intervention or control group) from a concealed list securely stored in the study coordinator’s office) | |
|  | 21b | Type of randomization (simple or restricted) and details of any factors for stratification. To reduce predictability of a random sequence, other details of any planned restriction (e.g., blocking) should be provided in a separate document that is unavailable to those who enroll participants or assign interventions | Page 7, lines 203–206 (without details on exact strata or block sizes) | |
| Allocation concealment  mechanism | 22 | Mechanism used to implement the random allocation sequence (e.g., central computer/telephone; sequentially numbered, opaque, sealed containers), describing any steps to conceal the sequence until interventions are assigned | See 21a for explanation | |
| Implementation | 23 | Whether the personnel who will enroll and those who will assign participants to the interventions will have access to the random allocation sequence | See 21a for explanation | |
| Blinding | 24a | Who will be blinded after assignment to interventions (e.g., participants, care providers, outcome assessors, data analysts) | Page 7, lines 207–208 | |
|  | 24b | If blinded, how blinding will be achieved and description of the similarity of interventions | Page 7, lines 207–210; see also 21a for explanation | |
|  | 24c | If blinded, circumstances under which unblinding is permissible, and procedure for revealing a participant’s allocated intervention during the trial | Page 7, lines 208–210 | |
| **Methods: Data collection, management, and analysis** | | | | |
| Data collection methods | 25a | Plans for assessment and collection of trial data, including any related processes to promote data quality (e.g., duplicate measurements, training of assessors) and a description of trial instruments (e.g., questionnaires, laboratory tests) along with their reliability and validity, if known. Reference to where data collection forms can be accessed, if not in the protocol | Pages 9–12, lines 263–318; Table 3 and Additional file 3 | |
|  | 25b | Plans to promote participant retention and complete follow-up, including list of any outcome data to be collected for participants who discontinue or deviate from intervention protocols | Pages 7–8, lines 211–219 | |
| Data management | 26 | Plans for data entry, coding, security, and storage, including any related processes to promote data quality (e.g., double data entry; range checks for data values). Reference to where details of data management procedures can be accessed, if not in the protocol | Page 7, lines 205–206 (further details on data management, including collection, storage, and data quality are provided in the full protocol versions) | |
| Statistical methods | 27a | Statistical methods used to compare groups for primary and secondary outcomes, including harms | Pages 13, lines 343–353 | |
|  | 27b | Definition of who will be included in each analysis (e.g., all randomized participants), and in which group | Page 12, lines 341–342 | |
|  | 27c | How missing data will be handled in the analysis | Page 13, lines 349–351 | |
|  | 27d | Methods for any additional analyses (e.g., subgroup and sensitivity analyses) | Page 13, lines 348–349 | |
| **Methods: Monitoring** | | | | |
| Data monitoring committee | 28a | Composition of data monitoring committee (DMC); summary of its role and reporting structure; statement of whether it is independent from the sponsor and funder; conflicts of interest and reference to where further details about its charter can be found, if not in the protocol. Alternatively, an explanation of why a DMC is not needed | Page 17, lines 480–487 | |
|  | 28b | Explanation of any interim analyses and stopping guidelines, including who will have access to these interim results and make the final decision to terminate the trial | Page 13, line 353; page 8, lines 220–221 | |
| Trial monitoring | 29 | Frequency and procedures for monitoring trial conduct. If there is no monitoring, give explanation | Page 17, lines 480–482 | |
| **Ethics** | | | | |
| Research ethics approval | 30 | Plans for seeking research ethics committee/institutional review board approval | Page 17, lines 468–475 | |
| Protocol amendments | 31 | Plans for communicating important protocol modifications to relevant parties | Page 17, lines 475–477 | |
| Consent or assent | 32a | Who will obtain informed consent or assent from potential trial participants or authorized proxies, and how | Page 17, lines 477–480; further details on responsible study team members and procedures are provided in the full protocol versions | |
|  | 32b | Additional consent provisions for collection and use of participant data and biological specimens in ancillary studies, if applicable | Not applicable, as no ancillary studies involving the collection or use of participant data or biological specimens are planned within ELIPSE I or II | |
| Confidentiality | 33 | How personal information about potential and enrolled participants will be collected, shared, and maintained in order to protect confidentiality before, during, and after the trial | Page 8, lines 236–237; and Additional file 3 (confidentiality and security measures regarding the apps); further details on data management, including collection, storage, and protection of participant are provided in the full protocol versions | |
| Ancillary and post-trial care | 34 | Provisions, if any, for ancillary and post-trial care, and for compensation to those who suffer harm from trial participation | Page 12, lines 326–327 | |

Citation: Chan A-W, Boutron I, Hopewell S, Moher D, Schulz KF, et al. SPIRIT 2025 statement: updated guideline for protocols of randomised trials. BMJ 2025;389:e081477. <https://dx.doi.org/10.1136/bmj-2024-081477>

© 2025 Chan A-W et al. This is an Open Access article distributed under the terms of the Creative Commons Attribution License (<https://creativecommons.org/licenses/by/4.0/>), which permits unrestricted use, distribution, and reproduction in any medium, provided the original work is properly cited.
