# Supplementary material for: Early Life Intervention in Paediatrics Supported by E-Health (ELIPSE)—a coaching app for parents to reduce obesity and second-hand smoke exposure in children: study protocols for two parallel-group randomised controlled trials
Source: Trials. 2025 Nov 18;26:520. doi: 10.1186/s13063-025-09251-5 (PMC12625043; doi:10.1186/s13063-025-09251-5)
Supplement: Supplementary file 2 — Additional file 2. SPIRIT figures. Schedule of assessments and procedures for ELIPSE I and II [file 13063_2025_9251_MOESM2_ESM.docx]

**Additional file 2** Schedule of assessments and procedures (SPIRIT figures) for ELIPSE I (A) and ELIPSE II (B).

| **ELIPSE I (obesity)** | **Time points** | | | | | | | | |
| --- | --- | --- | --- | --- | --- | --- | --- | --- | --- |
| **Procedures/Assessments** | **Baseline** | | | | **Intervention period**  weeks 2-22  (± 2 weeks) | **Post-intervention** | | **Follow-up** | |
|  | Approx.  -12 weeks | | **Visit 3  (day 0, app installation)** | Pre-evaluation  weeks 0-2  (± 1 week) |  | **Visit 4**  week 22  (-2/+4 weeks) | Post-evaluation  weeks 22-23  (± 4 weeks) | **Visit 5** week 48  (± 4 weeks) | Follow-up evaluation  weeks 48-49 (± 4 weeks) |
|  | Visit 1 | Visit 2 |  |  |  |  |  |  |  |
| Eligibility check | X |  |  |  |  |  |  |  |  |
| Written informed consent | X |  |  |  |  |  |  |  |  |
| Sociodemographic information | X |  |  |  |  |  |  |  |  |
| **Child-related medical endpoints^a^** |  |  |  |  |  |  |  |  |  |
| Parent questionnaire^b^ | X |  |  |  |  |  |  |  |  |
| Triaxial accelerometery | X |  |  |  |  | X^c^ |  | X^c^ |  |
| Nutritional assessment^d^ | X |  |  |  |  | X^c^ |  | X^c^ |  |
| Medical history | X |  |  |  |  |  |  |  |  |
| Family history | X |  |  |  |  |  |  |  |  |
| Clinical examination | X |  | (X)^e^ |  |  | X |  | X |  |
| Ambulatory blood pressure monitoring |  | X |  |  |  | X^c^ |  |  |  |
| Carotid-femoral PWV |  | X^c^ |  |  |  | X^c^ |  | X^c^ |  |
| Hand grip strength assessment | X |  | (X)^e^ |  |  | X |  | X |  |
| Fasting venous blood sample^f^ |  | X |  |  |  | X^c^ |  | X^c^ |  |
| **Parent-reported endpoints** |  |  |  |  |  |  |  |  |  |
| BIS-15 | X^g^ |  |  |  |  |  |  |  |  |
| DEBQ | X^g^ |  |  |  |  | X^h^ |  | X^h^ |  |
| CEBQ | X^g^ |  |  |  |  | X^h^ |  | X^h^ |  |
| PSS-10 | X^g^ |  |  |  |  |  |  |  |  |
| PHQ-9 | X^g^ |  |  |  |  |  |  |  |  |
| KIDSCREEN-27 | X^g^ |  |  |  |  | X^h^ |  | X^h^ |  |
| MTUAS | X^g^ |  |  |  |  |  |  |  |  |
| DERS-16 | X^g^ |  |  |  |  |  |  |  |  |
| DEAPQ-EL-GS | X^g^ |  |  |  |  | X^h^ |  | X^h^ |  |
| GAD-7 | X^g^ |  |  |  |  |  |  |  |  |
| Dietary habits (app-based) |  |  |  | X | X^i^ |  | X |  | X |
| Physical activity (app-based) |  |  |  | X | X^i^ |  | X |  | X |
| WAI-SR (app-based) |  |  |  |  | X^i^ |  |  |  |  |
| MAUQ |  |  |  |  |  | X^i^ |  |  |  |
| App follow-up questionnaire |  |  |  |  |  | X^i^ |  |  |  |
| **App-related endpoints** |  |  |  |  |  |  |  |  |  |
| Safety |  |  | X | X | X | X | X | X | X |
| App usability data collection |  |  |  | X | X^i^ |  | X |  | X |

**A**

^a^ Assessments are part of clinical routine except where otherwise indicated. (Continued on next page)

^b^ A questionnaire used to routinely collect medical history, socio-economic factors, and health behaviours from patients visiting the weight management service at the University Children’s Hospital Bern. Can be filled out until visit 3.
^c^ Study-related procedure/assessment for subjects participating in ELIPSE I that exceeds clinical routine in number of repetitions or extent (e.g., number of analysed blood parameters).
^d^ Nutritional assessments (24-hour recalls) are conducted following visits 1, 4 and 5. Recalls at baseline can be completed until end of pre-evaluation. In case of post-intervention and follow-up, recalls can be completed up to 2 weeks after end of post-evaluation and follow-up evaluation periods, respectively.
^e^ Visit 3 may be conducted either in person or by telephone. Assessments are optional if not scheduled as an in-person visit.
^f^ Venous blood sampling is routinely applied at baseline at the weight management service. For the purpose of this study, the full panel will be repeated at post-intervention and follow-up and the collected blood volume will be higher to account for biobank storage.
^g^ Questionnaires are sent following visit 1 or after inclusion/signing of informed consent and can be filled out until end of pre-evaluation period.
^h^ Post-intervention and follow-up questionnaires will be sent after visits 4 and 5, respectively, and can be filled out up to 2 weeks after end of post-evaluation and follow-up evaluation periods.
^i^ For intervention group only.
*BIS-15,* Barratt Impulsiveness Scale; *DEBQ,* Dutch Eating Behaviour Questionnaire; *CEBQ,* Children’s Eating Behaviour Questionnaire; *PSS-10,* Perceived Stress Scale; *PHQ-9,* Patient Health Questionnaire; *MTUAS* Media and Technology Usage and Attitudes Scale; *DERS-16,* Difficulties in Emotion Regulation Scale; *DEAPQ-EL-GS,* German extended version of the Alabama Parenting Questionnaire for primary school children; *GAD-7,* Generalized Anxiety Disorder Assessment; *WAI-SR,* Working Alliance Inventory short revised; *MAUQ,* mHealth App Usability Questionnaire.

| **ELIPSE II (smoke)** | **Time points** | | | | | | |
| --- | --- | --- | --- | --- | --- | --- | --- |
| **Procedures/ Assessments** | **Baseline** | | **Intervention period**  weeks 2-22  (± 2 weeks) | **Post-intervention** | | **Follow-up** | |
|  | **Baseline period weeks -4 to 0 (visit 1; day 0, app installation)** | Pre-evaluation  weeks 0-2 (± 1 week) |  | **Visit 2** week 22  (± 2 weeks) | Post-evaluation  weeks 22-23  (± 1 week) | **Visit 3** week 48  (± 4 weeks) | Follow-up evaluation  weeks 48-49 (± 1 week) |
| Eligibility check | X |  |  |  |  |  |  |
| Written informed consent | X |  |  |  |  |  |  |
| Sociodemographic information | X |  |  |  |  |  |  |
| **Child-related data** |  |  |  |  |  |  |  |
| Demographics | X |  |  |  |  |  |  |
| Medical history | X |  |  | X |  | X |  |
| Urine sample | X |  |  | X |  | X |  |
| **Parent-reported endpoints** |  |  |  |  |  |  |  |
| Demographics | X |  |  |  |  |  |  |
| Smoking behaviour^a^ | X | X | X^b^ | X | X | X | X |
| BIS-15 | X^c^ |  |  |  |  |  |  |
| PSS-10 | X^c^ |  |  |  |  |  |  |
| PHQ-9 | X^c^ |  |  |  |  |  |  |
| KIDSCREEN-27 | X^c,d^ |  |  | X^c^ |  | X^c^ |  |
| MTUAS | X^c^ |  |  |  |  |  |  |
| DERS-16 | X^c^ |  |  |  |  |  |  |
| DEAPQ-EL-GS | X^c^ |  |  | X^c^ |  | X^c^ |  |
| GAD-7 | X^c^ |  |  |  |  |  |  |
| WAI-SR (app-based) |  |  | X^b^ |  |  |  |  |
| MAUQ |  |  |  | X^b,c^ |  |  |  |
| App follow-up questionnaire |  |  |  | X^b,c^ |  |  |  |
| **App-related endpoints** |  |  |  |  |  |  |  |
| Safety | X | X | X | X | X | X | X |
| App usability data collection |  | X | X^b^ |  | X |  | X |

**B**

^a^ Smoking behaviour will be assessed using questionnaires at visits 1, 2, and 3. Additionally, app-based diary entries will be used to monitor smoking behaviour during the evaluation periods (and throughout the intervention period for the intervention group; see Supplementary file 3 for details).
^b^ For intervention group only.
^c^ Can be filled out until end of the respective evaluation period.
^d^ In ELIPSE II, an adapted short version of the KIDSCREEN questionnaire was used, as several items are not applicable to the younger age group. *BIS-15,* Barratt Impulsiveness Scale; *PSS-10,* Perceived Stress Scale; *PHQ-9,* Patient Health Questionnaire; *MTUAS* Media and Technology Usage and Attitudes Scale; *DERS-16,* Difficulties in Emotion Regulation Scale; *DEAPQ-EL-GS,* German extended version of the Alabama Parenting Questionnaire for primary school children; *GAD-7,* Generalized Anxiety Disorder Assessment; *WAI-SR,* Working Alliance Inventory short revised; *MAUQ,* mHealth App Usability Questionnaire.
