# Supplementary material for: Early Life Intervention in Paediatrics Supported by E-Health (ELIPSE)—a coaching app for parents to reduce obesity and second-hand smoke exposure in children: study protocols for two parallel-group randomised controlled trials
Source: Trials. 2025 Nov 18;26:520. doi: 10.1186/s13063-025-09251-5 (PMC12625043; doi:10.1186/s13063-025-09251-5)
Supplement: Supplementary file 3 — Additional file 3. Extended methods. 3a: Technical details of app development and platform. 3b: ELIPSE I (obesity) diary content. 3c: ELIPSE II (smoke) diary content. 3d: ELIPSE II (smoke) questionnaires on parental smoking behaviour and child respiratory health. Additional file 4. Ethical approval document. Confirmation by the Cantonal Ethics Committee of Berne, Switzerland. Additional file 5. Funding documentation. Confirmation of peer-review and funding information [file 13063_2025_9251_MOESM3_ESM.docx]

**Additional file 3** Extended methods.

**3a** Technical details of app development and platform.

The ELIPSE apps were developed at the University Hospital of Child and Adolescent Psychiatry and Psychotherapy Bern based on an existing mental health application platform. The apps are implemented cross-platform using React Native, an open-source framework developed by Meta (Meta Platforms, Inc., Menlo Park, USA) that uses JavaScript, making them available for both iOS and Android smartphones. All data generated in the app is processed in an anonymised manner to ensure that no personally identifiable information is captured.

On the server side, we use the Spring Boot framework, an open-source Java framework. The domain-specific code was implemented using a domain-specific programming language built on JetBrains Meta Programming System (JetBrains, Prague, Czech Republic), an open-source tool for creating custom programming languages and editors.

Access to the coach-backend is provided through an individually assigned access code for each coach, ensuring that only authorized personnel have access to the specific functions. Data is stored in a PostgreSQL database that is not accessible from outside; only the backend application can access the database, and it authorises all queries accordingly.

Communication between all systems (computers, mobile devices, or servers) is conducted via a TLS 1.2 secured HTTPS connection using an RSA certificate with a key length of 2048 bits. Additionally, the apps and server communicate over a Representational State Transfer Application Program Interface (REST API) that fully serialises the content of the apps, and push notifications are triggered by the server and delivered via the Apple Push Notification Service for iOS and Firebase Cloud Messaging for Android.

**3b** ELIPSE I (obesity) diary content. Participants are asked to complete daily app-based diary entries during the pre-, post-, and follow-up evaluation periods. For the intervention group, diary phases are repeated periodically throughout the intervention period.

| **Child’s diet** | |
| --- | --- |
| What type of meal would you like to record? | 1. Breakfast 2. Lunch 3. Dinner 4. Planned snack 5. Unplanned snack |
| Upload a photo of the meal or describe it! | [Photo or description] |
| How would you assess the portion size of your child's meal? | 1. Much too small 2. Slightly too small 3. Just right 4. Slightly too large 5. Much too large |
| How would you assess the nutritional balance of your child's meal? | 1. Very unbalanced 2. Rather unbalanced 3. Neither balanced nor unbalanced 4. Rather balanced 5. Very balanced |
| What did your child drink with the meal? | 1. Water 2. Sugary drinks 3. Fruit juices 4. Milk 5. Milkshake 6. Other drink 7. Nothing 8. Don’t know |
| **Child’s physical activity and screen time** | |
| How long was your child physically active today? | 1. 0 to 30 minutes 2. 30 to 60 minutes 3. 60 to 120 minutes 4. Over 120 minutes |
| How much screen time has your child had today? | 1. 0 to 30 minutes 2. 30 to 60 minutes 3. 60 to 120 minutes 4. Over 120 minutes |

**3c** ELIPSE II (smoke) diary content. Participants are asked to complete daily app-based diary entries during the pre-, post-, and follow-up evaluation periods. For the intervention group, diary phases are repeated periodically throughout the intervention period.

| **Parental smoking behaviour** | |
| --- | --- |
| How many cigarettes have you smoked today? | [answer] |
| How many of these cigarettes did you smoke in your child’s presence (regardless of location)? | [answer] |
| How many cigarettes did you smoke in places (e.g. home, car) where your child could or might be present? | [answer] |
| Which of the following protective measures did you take after smoking? | 1. Washed hands after smoking 2. Rinsed mouth after smoking 3. Avoided smoking in certain rooms 4. Changed clothes before or after smoking 5. Kept a distance from the child while smoking outdoors 6. None |

**3d** ELIPSE II (smoke) questionnaires on parental smoking behaviour and child respiratory health, as used at baseline, post-intervention and follow-up visits (adapted from REDCap and translated from German).

| **Parental smoking behaviour** | |
| --- | --- |
| Do you smoke every day? | 1. Yes 2. No 3. Quit smoking [only valid at post-intervention or follow-up] |
| If you quit smoking, when did you stop smoking? | [answer] |
| How many cigarettes do you smoke in a typical day? | 1. 5 or less 2. 6 to 15 3. 16 to 25 4. 26 to 35 5. more than 35 |
| How many years of your life have you smoked regularly?  *Asked only at baseline* | [answer] |
| How much time usually passes between waking up and smoking your first cigarette in the morning? | 1. 0-5 minutes 2. 6 to 30 minutes 3. 31 to 60 minutes 4. more than 60 minutes |
| Is your child currently being breastfed? | 1. Yes 2. No |
| If yes, is the mother participating in the study? | 1. Yes 2. No |
| If the mother is not a study participant, how many cigarettes does the mother smoke per day? | 1. Not at all 2. 5 or less 3. 6 to 15 4. 16 to 25 5. 26 to 35 6. More than 35 |
| How motivated are you to stop smoking at the moment? | 1. Not motivated at all 2. Not motivated 3. Hardly motivated 4. A little motivated 5. Quite motivated 6. Very motivated |
| How often have you tried to quit smoking in the past? | 1. Never 2. One to three times 3. Four to six times 4. Seven to ten times 5. More than ten times |
| Has the issue of second-hand smoking ever been addressed by your paediatrician?  *Asked only at baseline* | 1. Yes 2. No |
| If yes, how often was this discussed? | 1. Once 2. Mainly in the context of infections 3. Mainly as part of the regular checkups 4. Annually 5. At every appointment 6. Other [if selected, additional specification requested] |
| If yes, how was the topic of second-hand smoking discussed? *Multiple answers possible* | 1. Measures to reduce second-hand smoking explained 2. Information material handed out 3. Smoking cessation programmes recommended 4. A separate counselling appointment arranged 5. Other [if selected, additional specification requested] |
| Would you like to receive more information from your paediatrician about the topic of second-hand smoking?  *Asked only at baseline* | 1. Yes 2. No, I already feel sufficiently informed |
| If yes, what kind of information would you like to receive? | [answer] |
| Do you feel that you are able to apply your current knowledge to change your smoking behaviour? | 1. Yes 2. No |
| Which behaviours have you already been able to change? | [answer] |
| What kind of support would you need to make a behavioural change? | 1. A personal consultation with a specialist 2. An online coaching 3. A recommendation on which measures are most effective to protect my child from second-hand smoke 4. Other [if selected, additional specification requested] |
| When do you think the topic of second-hand smoking should be addressed? *Asked only at baseline* | 1. During routine check-ups 2. During infections 3. At every paediatric visit 4. Other [if selected, additional specification requested] |
| Would you be interested in a prevention clinic at a children’s hospital on the topic of ‘lung health’? | 1. Yes 2. No |
| Are you aware of the following measures to protect against second-hand smoking?  *Multiple answers possible* | 1. Washing your hands after smoking 2. Rinsing your mouth after smoking 3. Not smoking in certain rooms where the child is present (e.g. kitchen) 4. Not smoking in the car (if available). 5. Changing clothes after or before smoking 6. Keeping distance from the child while smoking outdoors 7. Other [if selected, additional specification requested] |
| How often do you use the measure ‘Wash hands after smoking’ while caring for your child? | 1. Never (0%) 2. Rarely (approx. 25%) 3. About half the time (approx. 50%) 4. Often (approx. 75%) 5. Always (100%) 8. Quit smoking [only valid at post-intervention or follow-up] |
| How often do you use the ‘Rinse mouth after smoking’ measure while caring for your child? | 1. Never (0%) 2. Rarely (approx. 25%) 3. About half the time (approx. 50%) 4. Often (approx. 75%) 5. Always (100%) 6. Quit smoking [only valid at post-intervention or follow-up] |
| How often do you apply the measure ‘Do not smoke in certain rooms where the child is (e.g. kitchen)’ while you are looking after your child? | 1. Never (0%) 2. Rarely (approx. 25%) 3. About half the time (approx. 50%) 4. Often (approx. 75%) 5. Always (100%) 6. Quit smoking [only valid at post-intervention or follow-up] |
| How often do you apply the measure ‘No smoking in the car (if available)’ while looking after your child? | 1. Never (0%) 2. Rarely (approx. 25%) 3. About half the time (approx. 50%) 4. Often (approx. 75%) 5. Always (100%) 7. Quit smoking [only valid at post-intervention or follow-up] |
| How often do you use the measure ‘Change clothes after or before smoking’ while looking after your child? | 1. Never (0%) 2. Rarely (approx. 25%) 3. About half the time (approx. 50%) 4. Often (approx. 75%) 5. Always (100%) 6. Quit smoking [only valid at post-intervention or follow-up] |
| How often do you apply the measure ‘Keep your distance from the child while smoking outdoors’ while looking after your child? | 1. Never (0%) 2. Rarely (approx. 25%) 3. About half the time (approx. 50%) 4. Often (approx. 75%) 5. Always (100%) 6. Quit smoking [only valid at post-intervention or follow-up] |
| Have you used any of the following other tobacco or nicotine products in the last 12 months?  *Multiple answers possible* | 1. Shisha 2. Cigars/cigarillos 3. Pipe 4. Snus or pouches 5. Snuff 6. Tobacco heater (IQOS or similar) 7. E-vaporiser (vape) 6. Other [if selected, additional specification requested] |
| How often have you used shisha? | 1. Daily 2. Several times a week 3. Once a week 4. Several times a month 5. Once a month 6. Less than once a month |
| How often do you use shisha per day? | [answer] |
| How often have you consumed cigars/cigarillos? | 1. Daily 2. Several times a week 3. Once a week 4. Several times a month 5. Once a month 6. Less than once a month |
| How often do you consume cigars/cigarillos per day? | [answer] |
| How often have you used a pipe? | 1. Daily 2. Several times a week 3. Once a week 4. Several times a month 5. Once a month 6. Less than once a month |
| How often do you use a pipe per day? | [answer] |
| How often have you used snus or pouches? | 1. Daily 2. Several times a week 3. Once a week 4. Several times a month 5. Once a month 6. Less than once a month |
| How often have you consumed snus or pouches? | [answer] |
| How often did you use snuff? | 1. Daily 2. Several times a week 3. Once a week 4. Several times a month 5. Once a month 6. Less than once a month |
| How often do you consume snuff per day? | [answer] |
| How often have you used tobacco heaters? | 1. Daily 2. Several times a week 3. Once a week 4. Several times a month 5. Once a month 6. Less than once a month |
| How often do you use tobacco heaters per day? | 7. [answer] |
| How often have you used e-vaporisers (vape)? | 1. Daily 2. Several times a week 3. Once a week 4. Several times a month 5. Once a month 6. Less than once a month |
| How often do you use e-vaporisers (vape) per day? | [answer] |
| How many smokers live in your household (without you)? | [answer] |
| If other smokers, who is this person? *Question repeats for each additional smoker living in the household* | [answer] |
| Does this person smoke inside the home? *Question repeats for each additional smoker living in the household* | 1. Yes 2. No |
| Approximately how many cigarettes does this person smoke per day? *Question repeats for each additional smoker living in the household* | [answer] |
| What percentage of your circle of friends are smokers? | 1. 0% 2. 1-25% 3. 26-50% 4. 51-75% 5. 76-100% |
| Have you used the following substances in the last 12 months? | 1. Alcohol 2. Cannabis 3. Cocaine 4. Ecstasy 5. MDMA 6. LSD 7. Other substances |
| How often have you consumed alcohol? | 1. Daily 2. Several times a week 3. Once a week 4. Several times a month 5. Once a month 6. Less than once a month |
| How many standard drinks do you drink per day (1 standard drink = 3 dL beer, 1 dL wine, 3cl schnapps) | [answer] |
| How often have you used cannabis? | 1. Daily 2. Several times a week 3. Once a week 4. Several times a month 5. Once a month 8. Less than once a month |
| How many joints of cannabis do you smoke per day? | [answer] |
| How often have you used cocaine? | 1. Daily 2. Several times a week 3. Once a week 4. Several times a month 5. Once a month   Less than once a month |
| How many lines of cocaine do you consume per day? | 6. [answer] |
| How often have you used ecstasy? | 1. Daily 2. Several times a week 3. Once a week 4. Several times a month 5. Once a month   Less than once a month |
| How often do you consume ecstasy per day? | 6. [answer] |
| How often have you used MDMA? | 1. Daily 2. Several times a week 3. Once a week 4. Several times a month 5. Once a month   Less than once a month |
| How often do you consume MDMA per day? | 6. [answer] |
| How often have you used LSD? | 1. Daily 2. Several times a week 3. Once a week 4. Several times a month 5. Once a month   Less than once a month |
| How often do you consume LSD per day? | 6. [answer] |
| What other substances? | [answer] |
| How often have you used other substances? | 1. Daily 2. Several times a week 3. Once a week 4. Several times a month 5. Once a month 6. Less than once a month |
| How often do you consume other substances per day? | [answer] |
| Is your child looked after by someone else who smokes? | 1. Yes 2. No |
| How many hours per week is your child looked after by this/these person(s)? | [answer] |
| **Child respiratory health** | |
| In the last 12 months (or since last visit), how many times has your child had a runny nose, cough or cold? | [answer] |
| In the last 12 months (or since last visit), how many times has your child had a tonsillitis or sore throat? | [answer] |
| In the last 12 months (or since last visit), how many times has your child had a pneumonia? | [answer] |
| In the last 12 months (or since last visit), how many times has your child experienced wheezing? | [answer] |
| In the last 12 months (or since last visit), how many times has your child had earache or a middle ear infection? | [answer] |
| How often has your child been to the doctor in the last 12 months (or since last visit) due to one or more of the above symptoms? | [answer] |
| How often has your child been hospitalised in the last 12 months (or since last visit) due to one or more of the above symptoms (including overnight stays)? | [answer] |
| How often has your child had to take antibiotics for pneumonia in the last 12 months (or since last visit)? | [answer] |
| How often has your child had to take antibiotics for tonsillitis in the last 12 months (or since last visit)? | [answer] |
| How often has your child had to take antibiotics for middle ear infection in the last 12 months (or since last visit)? | [answer] |
| How often has your child had to take antibiotics for another cause in the last 12 months (or since last visit)? | [answer] |
| If other cause, please specify. | [answer] |
| Has your child had to inhale (e.g. with salbutamol) in the last 12 months (or since last visit) due to respiratory symptoms? | 1. No 2. Yes, several days/weeks at a time 3. Yes, occasionally if required 4. Yes, as permanent therapy |
| How many episodes of respiratory illness with continuous inhalation have there been in the last 12 months (or since last visit)? | [answer] |
| What was the average number of days of inhalation in these episodes? | [answer] |
| In the case of on-demand medication, how often was inhaled? | 1. Several times a week 2. Once a week 3. Several times a month 4. Once a month 5. Occasionally in the winter months 5. Less often |
|  |  |
|  |  |
